# Diagnostic Values of Free Triiodothyronine and Free Thyroxine and the Ratio of Free Triiodothyronine to Free Thyroxine in Thyrotoxicosis

**DOI:** 10.1155/2018/4836736

**Published:** 2018-06-04

**Authors:** Xinxin Chen, Yulin Zhou, Mengxi Zhou, Qinglei Yin, Shu Wang

**Affiliations:** Shanghai Clinical Center for Endocrine and Metabolic Diseases, Department of Endocrinology, Ruijin Hospital, Shanghai Jiao Tong University School of Medicine, 197 Ruijin 2nd Road, Shanghai 200025, China

## Abstract

**Background:**

The results of previous studies on the usefulness of free triiodothyronine (FT3) to free thyroxine (FT4) are controversial. We investigated the usefulness of FT3, FT4, and FT3/FT4 ratio in differentiating Graves' disease (GD) from destructive thyroiditis.

**Methods:**

A total of 126 patients with untreated GD, 36 with painless thyroiditis, 18 with painful subacute thyroiditis, and 63 healthy controls, were recruited. The levels of FT3 and FT4 and the FT3/FT4 ratios for the different etiologies of thyrotoxicosis were evaluated separately by receiver operating characteristic (ROC) curve analysis. The expression levels of type 1 and type 2 deiodinase (*DIO1* and *DIO2*) in thyroid tissues were also investigated.

**Results:**

The optimal cut-off values were 7.215 pmol/L for FT3, 21.71 pmol/L for FT4, and 0.4056 for the FT3/FT4 ratio. The specificity and positive predictive value of the FT3/FT4 ratio were highest for values > 0.4056. *DIO1* mRNA expression was significantly higher in the thyroid tissue of patients with GD (*P* = 0.013).

**Conclusions:**

We demonstrated that the FT3/FT4 ratio was useful in differentiating GD from destructive thyroiditis. In addition, a relatively high expression of type 1 deiodinase in the thyroid might be responsible for the high FT3/FT4 ratio in patients with GD.

## 1. Introduction

Thyrotoxicosis can occur if thyroid hormone is passively released in excessive amounts or if the thyroid follicle cells are constitutively activated for thyroid hormone synthesis and secretion. Hyperthyroidism is generally considered to be caused by the sustained overproduction and release of hormone by the thyroid itself. Graves' disease (GD) is the most common etiologies of hyperthyroidism, characterized by circulating antibodies to the thyrotropin receptor (TRAb) [1, 2]. Due to thyroid follicle cell breakdown from an autoimmune reaction, infection, drugs, painless thyroiditis, and subacute thyroiditis, also known as destructive thyroiditis, are less common causes of thyrotoxicosis. Destructive thyroiditis usually manifests as transient thyrotoxicosis followed by the recovery of thyroid function or the development of transient or permanent thyroid failure.

It is important to differentiate the etiologies of thyrotoxicosis. For example, hyperthyroidism patients with GD must be treated with antithyroid drugs, radioisotope therapy, or surgery, which are unnecessary for patients with destructive thyroiditis [3]. The clinical diagnosis of patients with thyrotoxicosis is generally based on the physical manifestations and laboratory test results. Characterized by fever and thyroid pain, subacute thyroiditis is not difficult to diagnose. However, it is difficult to differentiate GD from painless thyroiditis based on the physical manifestations. The radioactive iodine (RAI) uptake test is a useful method to differentiate thyrotoxicosis etiologies; however, it is contradicted in specific situations such as in pregnant or lactating women. The postpartum period is the most common time for painless thyroiditis to appear [3]. In addition, exposure to large amounts of iodine prior to undergoing the RAI uptake test may suppress RAI uptake, resulting in an incorrect diagnosis. Detection of TRAb has become the most commonly used method to differentiate the etiology of thyrotoxicosis when RAI uptake is unavailable or contraindicated [3]. A third-generation assay for TRAb, which is based on the detection of immunosorbent biotin-streptavidin binding of human monoclonal thyroid-stimulating antibodies to recombinant thyroid-stimulating hormone (TSH), has been developed, offering a sensitivity of 95% and 100% specificity [4]. However, patients with mild GD might be negative for TRAb. A practical alternative for differentiating diagnosis was in need in clinic.

The ratio of serum triiodothyronine (T3) to thyroxine (T4) levels has been recommended as a useful indicator for differentiating patients with GD from those with destructive thyroiditis such as painless thyroiditis [3]. Theoretically, T3 levels will be elevated above the upper limit of the normal range more than those of T4 due to the hyperfunction of thyroid itself, whereas the ratio of T3/T4 might not be changed due to thyroid hormone released by destructive thyroiditis. In one study, the ratio of total T3 to total T4 was useful for the differentiation of patients with GD from those with postpartum thyroiditis [5]. The measurement of total form T3 and T4 was affected by thyroid hormone-binding proteins. The measurement of FT3 and FT4 was less affected and used for the differentiation and diagnosis of GD and destructive thyroiditis [6]. However, some studies have found that the FT3 to FT4 ratio is not useful for differentiating thyrotoxicosis etiologies [7].

Therefore, the present study evaluated the usefulness of FT3 and FT4 levels and the FT3/FT4 ratio in the differentiating patients with GD from those with destructive thyroiditis, especially those with painless thyroiditis and subacute thyroiditis.

## 2. Patients and Methods

### 2.1. Patients

Patients were recruited from Ruijin Hospital affiliated to Shanghai Jiao Tong University School of Medicine since March 2016. Patients with different etiologies of thyrotoxicosis were recruited to participate in this study. GD was defined based on clinical symptoms, including heat intolerance, fatigue, increased appetite, increased sweating, weight loss, muscle weakness, tremors, and diffusely enlarged thyroid gland. Laboratory diagnosis included increased serum concentrations of FT4 and/or FT3, decreased basal TSH level, and positive TRAb findings. Thyrotoxicosis patients negative for TRAb but with diffuse high RAI uptake were also confirmed to have GD. Patients with GD were naïve to any treatment at the time of diagnosis. Healthy controls and thyrotoxicosis patients with painless thyroiditis and subacute thyroiditis were also recruited. The healthy control had no personal or family history of thyroid disease; had normal thyroid ultrasound imaging; serum TSH values and FT3/FT4 ratio within reference ranges; and were negative for thyroid peroxidase antibodies (TPOAb), thyroglobulin antibodies (TgAb), and TRAb. Patients with painless thyroiditis and subacute thyroiditis were recruited at the initial time of diagnosis in this study, which was characterized by low levels of TSH and high levels of FT3 and FT4 in the serum. Painless thyroiditis was defined as thyrotoxicosis with low RAI uptake, with or without the presence of increased serum level of antibodies to TPOAb and/or positive for TgAb. Subacute thyroiditis was diagnosed based on clinical findings of severe neck pain, fever, thyrotoxicosis, elevated erythrocyte sedimentation rate (ESR), and low RAI uptake.

### 2.2. Laboratory Tests

Serum levels of TSH, FT3, FT4, TgAb, and TPOAb were measured using automated chemiluminescent immunoassays (Architect i2000SR; Abbott Laboratories, Chicago, IL). The functional sensitivity of serum TSH was 0.0036 mIU/L. The intra-assay coefficients of variation (CV) of serum TSH, FT4, FT3, TPOAb, and TgAb were 1.3–6.3% and the interassay CV values were 2.0%–6.6%. The laboratory reference ranges provided by the manufacturer used in this study were as follows: TSH 0.35–4.94 *μ*IU/mL, FT4 9.01–19.04 pmol/L, FT3 2.63–5.70 pmol/L, TPOAb < 5.61 IU/ml, and TgAb < 4.11 IU/mL. Serum levels of TRAb were measured by electrochemiluminescence immunoassays (Cobas 601 analyzer, Roche Diagnostics) with a suggested cut-off value of 1.75 IU/L. The functional sensitivity of serum TRAb was 0.3 IU/L. The laboratory reference range for ESR was <20 and <15 mm/h for women and men, respectively. Clinical data on RAI uptake were obtained at the nuclear medicine department of Ruijin Hospital affiliated to Shanghai Jiao Tong University School of Medicine. The reference range for RAI uptake was 10–30% for 3-hour uptake and 45–60% for 24-hour uptake.

### 2.3. Thyroid Tissues

We also collected thyroid tissue samples from 10 GD patients who had undergone thyroidectomy between April 2017 and October 2017 at Ruijin Hospital. The GD diagnosis was before surgery. At the time of surgery, the GD patients were euthyroid following methimazole therapy. Normal thyroid tissue was taken from patients who had undergone thyroidectomy due to thyroid nodules. Six samples of normal thyroid tissue were collected from tissues adjacent to the thyroid nodule and confirmed by pathological examination.

This study was approved by the institutional review board of the Ruijin Hospital affiliated to Shanghai Jiao Tong University School of Medicine. The written informed consent was obtained from each participant. The study was in accordance with the principle of the Helsinki Declaration II. All study participants provided written informed consents.

### 2.4. RNA Preparation and Real-Time Quantitative Polymerase Chain Reaction (PCR)

Total RNA from thyroid tissues was isolated using the TRIzol reagent (Invitrogen) according to the manufacturer's protocol. One microgram total RNA was converted into first-strand cDNA using a commercial first-strand cDNA synthesis kit (Promega, Madison, WI, USA) according to the manufacturer's instructions. Real-time quantitative PCR was performed using SYBR master mix (Takara, Shiga, Japan) on a LightCycler® 480 system (Roche, Pleasanton, CA, USA). The human *GAPDH* gene was used as an endogenous control for sample normalization. The results were presented as the fold expression relative to that of *GAPDH*. The PCR primers were as follows: for human *GAPDH*, forward 5′-TGATGACATCAAGAAGGTGGTGAAG-3′ and reverse 5′-TCCTTGGAGGCCATGTGGGCCAT-3′; for human *DIO1*, forward 5′ -TTAGTTCCATAGCAGATTTTCTTGTCA-3′ and reverse 5′-CTGATGTCCATGTTGTTCTTAAAAGC-3′; and for human *DIO2*, forward 5′-TCA- TTCTGCTCAAGCACGTG-3′ and reverse 5′-ACCATTGCCACTGTTGTCAC-3′.

### 2.5. Statistical Analysis

All statistical analyses were performed using IBM SPSS Statistics for Windows, version 19.0 (IBM Corp., Armonk, NY, USA). All graphics were produced using *GRAPH PAD PRISM 6.0* (GraphPad Software, La Jolla, CA, USA). Descriptive data were shown as mean ± SD for normally distributed continuous parameters, and for skewness distribution data, median and interquartile range was used. *P* < 0.05 were considered statistically significant. Correlations between the different variables were analyzed by correlation analysis using *Pearson's tests*. Differences between multiple groups were tested by one-way analysis of variance (*ANOVA*), and post hoc comparisons were performed using *Tukey's tests*. A receiver operating characteristic (ROC) curve analysis was performed to obtain the optimal cut-off values for FT3, FT4, and FT3/FT4 ratio for the diagnosis of GD. The sensitivity and specificity of FT3 and FT4 level and FT3/FT4 ratio were estimated from the ROC curves. The positive predictive values (PPVs) and negative predictive values (NPVs) from the ROC curves for FT3, FT4, and the FT3/FT4 ratio were also calculated and compared.

## 3. Results

### 3.1. Clinical Characteristics of the Subjects

The demographic and clinical data of the study population are shown in Table 1. In total, we recruited 126 untreated GD patients (40.25 ± 12.49 years), 92.8% (117/126) of whom were TRAb positive. 7.2% (9/126) of patients who were TRAb-negative were confirmed to have GD based on a typical “thyroid storm pattern” in ultrasound imaging of the neck and diffuse increase in RAI uptake. Thirty-six patients had painless thyroiditis (30 females, mean age 40.00 ± 12.79 years), 18 had painful subacute thyroiditis (13 females, mean age 43.94 ± 7.36), and 63 healthy control (49 females, mean age 48.05 ± 14.72 years) were also recruited in our study. All patients with subacute thyroiditis had a high ESR (mean value 66.53 ± 21.19 mm/h).

### 3.2. FT3 and FT4 Levels and FT3/FT4 Ratio in Different Thyrotoxicosis Etiologies

**Figure 1:**
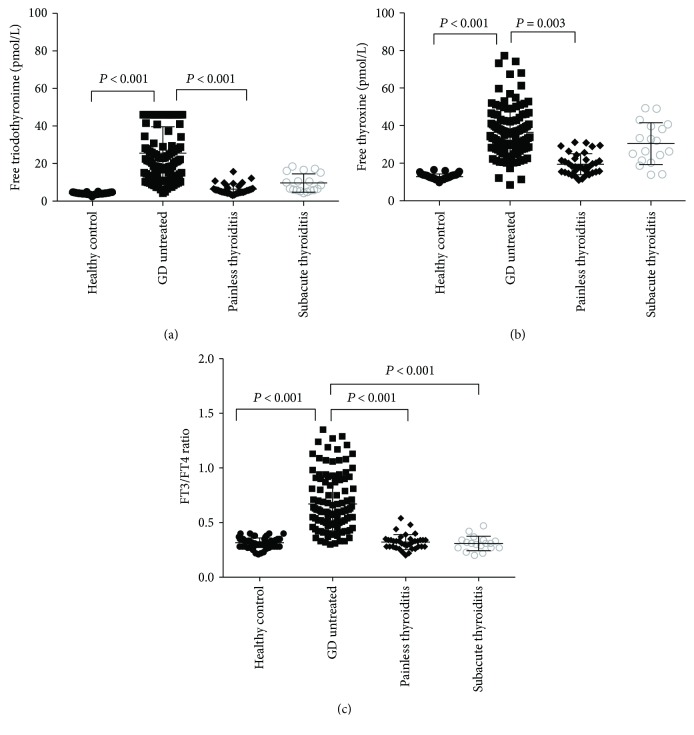
FT3, FT4, and FT3/FT4 ratios in different groups of patients with thyrotoxicosis at initial of diagnosis and in healthy controls. (a) shows serum free triiodothyronine (FT3) levels. (b) shows serum free thyroxine (FT4) levels. (c) shows FT3/FT4 ratios in different groups of patients with thyrotoxicosis at initial of diagnosis and in healthy controls.

All untreated GD patients had higher FT3 and FT4 levels and FT3/FT4 ratio and lower TSH levels. As shown in Figures 1(a) and 1(b), FT3 and FT4 concentrations were significantly higher in untreated GD compared to those in healthy controls and patients with painless thyroiditis. However, the FT3 and FT4 levels did not differ significantly between patients with untreated GD and those with subacute thyroiditis. The TRAb level was highest in the untreated GD group (*P* < 0.001). There was no significant difference in TPOAb and TgAb levels between the untreated GD and painless thyroiditis groups.

As shown in Figure 1(c), the FT3/FT4 ratio in untreated GD (0.66 ± 0.26) was significantly higher than those in the painless thyroiditis (0.32 ± 0.07, *P* < 0.001) and subacute thyroiditis (0.31 ± 0.07, *P* < 0.001) groups. The FT3/FT4 ratio did not differ significantly between the painless thyroiditis (0.32 ± 0.07), healthy control (0.32 ± 0.04), and subacute thyroiditis (0.31 ± 0.07) groups.

**Figure 2:**
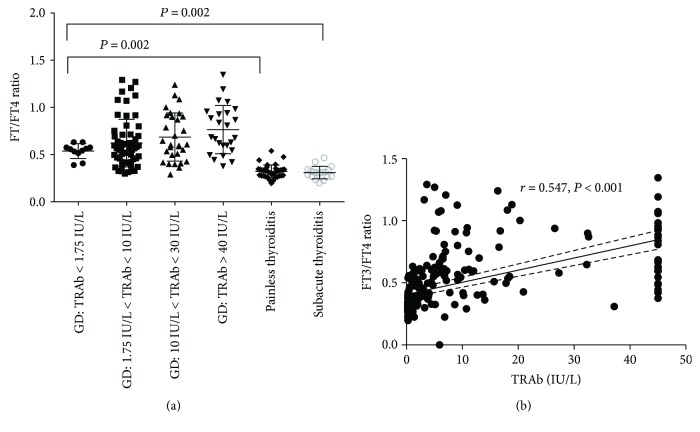
Correlation between FT3/FT4 ratio and TRAb. (a) shows FT3/FT4 ratios for different TRAb titers in patients with Graves' disease as well as the FT3/FT4 ratios for patients with painless thyroiditis and subacute thyroiditis. (b) shows correlation between FT3/FT4 ratio and TRAb titer.

We then analyzed the FT3/FT4 ratios in untreated GD patients grouped by TRAb levels. As shown in Figure 2(a), the ratio increased with increasing TRAb tilters in untreated GD patients. The FT3/FT4 ratio in the TRAb-negative GD group was higher than those in the painless thyroiditis (*P* = 0.002) and subacute thyroiditis (*P* = 0.002) groups. Correlation analysis showed that the FT3/FT4 ratio was positively correlated with TRAb level (*r* = 0.547, *P* < 0.001, Figure 2(b)).

### 3.3. Diagnostic Value of FT3, FT4, and FT3/FT4 Ratio in Patients with Untreated GD

**Figure 3:**
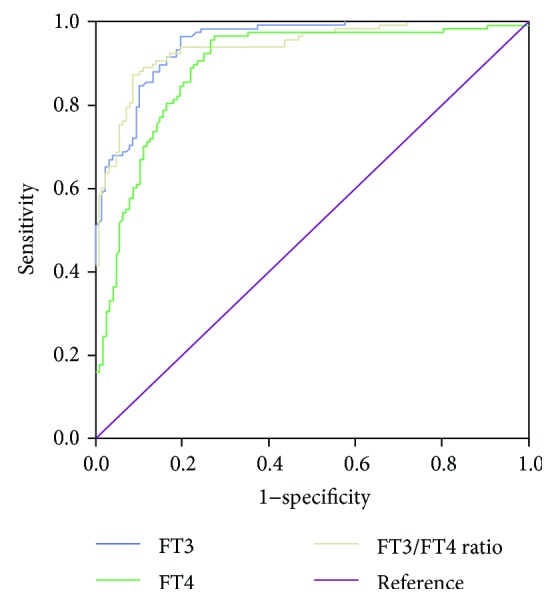
Receiver operating characteristic (ROC) curve for the discrimination of patients with Graves' disease from healthy controls and patients with destructive thyroiditis.

To obtain the optimal diagnostic cut-off value of FT3, FT4, and FT3/FT4 ratio, we performed ROC curve analysis of all untreated thyrotoxicosis patients and 63 healthy controls, as shown in Figure 3. For FT3, the optimal cut-off value was 7.215 pmol/L, with a sensitivity and specificity of 96.6% and 80.5%, respectively, with an area under the curve (AUC) of 0.949 (95% confidence interval (CI): 0.925–0.973). For FT4, the optimal cut-off value was 20.71 pmol/L with a sensitivity of 96.6%, specificity of 72.7%, and AUC of 0.849 (95% CI: 0.852–0.935). The optimal FT3/FT4 ratio cut-off value was 0.4056, with a sensitivity of 87.3%, specificity of 91.4%, and AUC of 0.940 (95% CI: 0.912–0.969).

Twenty-five percent (9/36) of patients with painless thyroiditis and 50% (9/18) of those with subacute thyroiditis had FT3 > 7.251 pmol/L, while 36.1% (13/36) of patients with painless thyroiditis and 83.3% (15/18) of those with subacute thyroiditis had FT4 > 20.71 pmol/L. Only 5.56% (2/36) of patients with painless thyroiditis and 11.1% (2/18) of those with subacute thyroiditis had FT3/FT4 ratios > 0.4056. Using the optimal cut-off value based on ROC analysis, the positive predictive values (PPVs) and negative predictive values (NPVs) were calculated, as shown in Table 2. The specificity and PPV of the FT3/FT4 ratio were highest compared to those of both FT3 and FT4 at the best cut-off values.

### 3.4. *DIO1* and *DIO2* mRNA Levels in Thyroid Tissue

**Figure 4:**
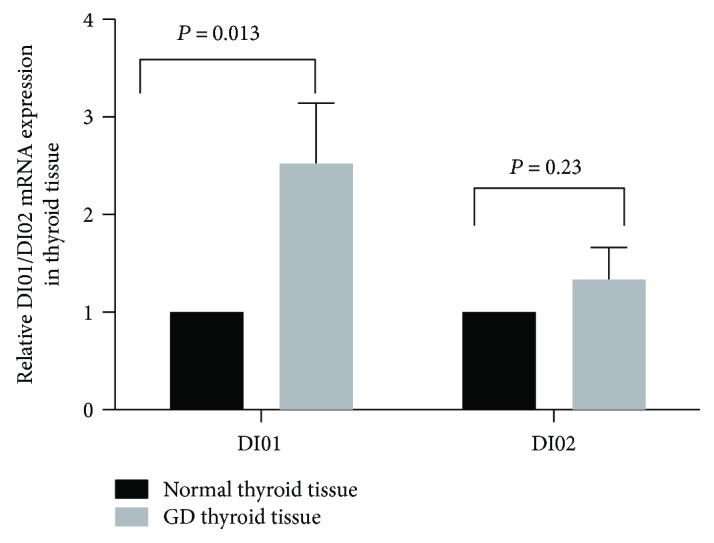
Relative DIO1/DIO2 mRNA expression levels in the thyroid tissue of Graves' disease and normal thyroid tissue.

We investigated the *DIO1* and *DIO2* mRNA expression in GD and normal thyroid tissues. The real-time quantitative PCR results showed that the expressions of *DIO1* and *DIO2* mRNA were higher in GD thyroid tissue than that in normal thyroid tissue (Figure 4). Especially, *DIO1* mRNA in GD thyroid tissue was significantly higher than that in normal thyroid tissue (*P* = 0.013).

## 4. Discussion

Multiple etiologies may contribute to thyrotoxicosis. The initial evaluation of the etiology is crucial to the appropriate clinical management of thyrotoxicosis. Although the clinical presentation of thyrotoxicosis may provide clues for diagnosis, it is not always reliable for the assessment of the etiology of thyrotoxicosis. Apart from the typical clinical presentation, measurement of serum levels of TRAb, RAI uptake, or measurement of thyroidal blood flow on ultrasonography are commonly used. However, RAI uptake and measurement of thyroidal blood flow are complicated procedures and not always available. In addition, RAI uptake is contraindicated in pregnant or lactating women. The postpartum period is the most common time when painless thyroiditis is observed [3]. It is hard to differentiate painless thyroiditis and GD without RAI uptake. Patients with overt thyrotoxicosis should undergo biochemical evaluations by measurement of serum levels of TSH, total T3, and FT4. More information may be available with a better understanding of the distributions and characteristics of thyroid hormones in different thyrotoxicosis etiologies.

The ratio of total T3 and total T4 levels are reportedly useful in differentiating the etiology of thyrotoxicosis. One study investigated the hormone presentations of different thyrotoxicosis etiologies. The results showed that the T3/T4 ratio was higher in patients with GD than those in patients with subacute thyroiditis and painless thyroiditis, with proposed T3/T4 ratios (ng/*μ*g) of >20 for GD and toxic nodular goiter and <20 for painless or postpartum thyroiditis [5]. Total T3 and total T4 levels are affected by thyroxine-binding globulin (TBG) concentration. Serum free T3 and free T4 levels are less affected. Several studies further evaluated the usefulness of the FT3/FT4 ratio for the differential diagnosis of GD. Shigemasa et al. found that the FT3/FT4 ratio was not useful in differentiating GD from painless thyroiditis [6]. However, Izumi et al. reported that the FT3/FT4 ratio was useful in differentiating GD and destruction-induced thyrotoxicosis, although some overlap was observed [7]. According to Yoshimura et al., the FT3/FT4 ratio might be useful in differentiating patients with GD and painless thyroiditis when the FT4 value was high [8]. In addition, Sriphrapradang et al. reported that the FT3/FT4 ratio might help in differentiating the cause of thyrotoxicosis, with a higher FT3/FT4 ratio suggesting GD, whereas a very low ratio supported the diagnosis of subacute thyroiditis [9].

Painless thyroiditis and subacute thyroiditis are most common etiologies of destructive thyroiditis, characterized by the leakage of thyroid hormone from the breakdown thyroid follicle cell. In our study, we analyzed levels of FT3 and FT4 and FT3/FT4 ratio in patients with GD and destructive thyroiditis, especially subacute thyroiditis and painless thyroiditis. We found that the levels of FT3 and FT4 and the FT3/FT4 ratio were highest in patients with untreated GD. Consistent with previous reports [7–9], we noticed an overlap among the different thyrotoxicosis etiologies of patients in our study. However, the FT3/FT4 ratio in GD had less overlapped with painless thyroiditis and painful subacute thyroiditis compared to those of the FT3 and FT4 concentrations. To obtain an optimal cut-off value, we performed ROC curve analysis. The results showed that FT3 > 7.251 pmol/L, FT4 > 20.71 pmol/L, and FT3/FT4 ratio > 0.4056 were more likely to have GD. We also compared the PPV and NPV for the diagnosis of GD using the optimal cut-off values for FT3, FT4, and FT3/FT4 ratio. The results showed that the specificity and PPV were highest for FT3/FT4 ratio > 0.4056 when compared to those of FT3 or FT4. Thus, the FT3/FT4 ratio might be more suitable for the differential diagnosis of the different etiologies of thyrotoxicosis. This conclusion was further confirmed in the group of TRAb-negative GD patients. Nine patients with thyrotoxicosis were confirmed to have GD based on diffuse high RAI uptake or typical “thyroid storm pattern” in ultrasound imaging of the neck and negative TRAb findings at initial diagnosis. In the current study, the FT3/FT4 ratio was 0.51 ± 0.09 in the TRAb-negative GD group, significantly higher than that in patients with painless thyroiditis and subacute thyroiditis. Eight of the 9 TRAb-negative GD patients had FT3/FT4 ratios > 0.4056. TSH-binding inhibition assays (TBII) and bioassays are currently the most common techniques for the measurement of TRAb levels. However, some patients with mild GD might test negative for TRAb, which complicates the assessment of the etiology of thyrotoxicosis. In that case, the FT3/FT4 ratio was useful in differentiating patients with GD from those with destructive thyroiditis and could serve as a practical alternative.

Due to the hyperactive thyroid gland, more T3 than T4 is produced. The mechanism is related to thyroidal deiodinases. Only 20% circulating T3 is reportedly secreted by the thyroid in euthyroid individuals and 80% of circulating T3 originates from the deiodination of T4 in the peripheral blood [10]. However, the percent is up to two-thirds higher in patients with hyperthyroidism, with the majority produced by the deiodination of T4 in the thyroid [11]. The production of T3 from the thyroid originates from the deiodination of T4 in the thyroid and from the hydrolysis of thyroglobulin. There are two types of deiodinases in thyroid, type 1 and type 2 (*DIO1* and *DIO2*) [12, 13]. Both DIO1 and DIO2 are present in the human thyroid. The roles of DIO1 and DIO2 in the production of circulating T3 in hyperthyroidism patients is controversial. Salvatore et al. found that intrathyroidal DIO2 might contribute to the relative increase of T3 production in patients with GD [14]. An indirect method using propylthiouracil (PTU) in that study showed that DIO1-generated T3 in the thyroid gland was the major source of plasma T3 in patients with hyperthyroidism since PTU could inhibit DIO1 [11]. We, therefore, investigated the relative expressions of thyroidal *DIO1* and *DIO2*. The results showed that both *DIO1* and *DIO2* levels were increased in the thyroid tissue of patient with GD; in particular, *DIO1* levels were significantly higher than those in the control tissue, which was consistent with findings reported by Laurberg et al. [11]. Ito et al. found a strong positive correlation between thyroidal *DIO1* and *DIO2* mRNA levels and serum TRAb titers, suggesting that the increment of thyroidal DIO1 and especially DIO2 might be responsible for the higher serum FT3/FT4 ratio in T3-predominant GD [15]. *DIO1* mRNA is reportedly positively regulated by cAMP, while *DIO2* is negatively regulated by T3 and positively regulated by cAMP [16–18]. Although we do not have direct results to indicate which deiodinase is responsible for the elevated serum T3 level in patients with GD, the *DIO1* mRNA expression level increased more significantly than that of *DIO2* mRNA; an enhanced *DIO1* might at least partly contribute to the higher FT3/FT4 ratio in patients with GD. Further study is necessary to confirm and clarify these specific mechanisms.

## 5. Conclusions

The results of this study demonstrated that the FT3/FT4 ratio overlapped less for different etiologies of thyrotoxicosis and was useful for the differentiation of GD from destructive thyroiditis, including painless thyroiditis and subacute thyroiditis. For patients with mild GD or who are unable to undergo the RAI uptake test, the FT3/FT4 ratio is a simple and practical alternative to assess the etiology of thyrotoxicosis. Increased thyroidal *DIO1* mRNA levels might contribute to the higher FT3/FT4 ratio in patients with GD.

## Figures and Tables

**Table 1:** Clinical characteristics of the study population.

	Healthy control	Untreated Graves' disease	Painless thyroiditis	Subacute thyroiditis
Gender (M/F)	17/49	25/101	6/30	5/13
Age (years)	48.05 ± 14.72	40.25 ± 12.49	40.00 ± 12.79	43.94 ± 7.36
FT3 (pmol/L)	4.04 ± 0.50	24.85 ± 14.02^ab^	6.36 ± 2.74	9.69 ± 4.88
FT4 (pmol/L)	12.87 ± 1.39	36.26 ± 14.09^ab^	19.48 ± 5.64	30.49 ± 11.08
FT3/FT4 ratio	0.32 ± 0.04	0.66 ± 0.26^abc^	0.32 ± 0.07	0.31 ± 0.07
TSH (mIU/L)	1.83 (0.54–3.25)	0.0006 (0.0001–0.01)^a^	0.01 (0.0001–0.2927)	0.0046 (0.0001–0.39)
TPOAb (IU/L)	0.43 ± 0.59	444.33 ± 393.43^ac^	336.45 ± 380.16	2.29 ± 3.59
TgAb (IU/L)	14.19 ± 87.19	221.29 ± 290.78^ac^	207.87 ± 292.53	18.54 ± 31.78
TRAb (IU/L)	0.33 ± 0.08	15.51 ± 16.15^abc^	0.60 ± 0.45	2.81 ± 8.71

Descriptive data were shown as mean ± SD for normally distributed continuous parameters, and for skewness distribution data, median and interquartile range was used. *P* < 0.05 were considered statistically significant. Differences between multiple groups were tested by one-way analysis of variance (*ANOVA*) and post hoc comparisons were performed using *Tukey's tests*. ^a^Significance difference between untreated GD and healthy control at *P* < 0.001. ^b^Significance difference between untreated GD and painless thyroiditis at *P* < 0.001. ^c^Significance difference between untreated GD and subacute thyroiditis at *P* < 0.001.

**Table 2:** Diagnostic evaluation of cut-off levels of FT3, FT4, and FT3/FT4 ratio in untreated Graves' disease.

	FT3 > 7.215 pmol/L	FT4 > 20.71 pmol/L	FT3/FT4 > 0.4056
PPV	122/140 (87.14%)	121/149 (81.21%)	109/113 (96.46%)
NPV	4/10 (10.00%)	5/31 (16.13%)	17/66 (25.76%)
Sensitivity	96.60%	96.60%	87.30%
Specificity	80.50%	72.70%	91.40%
AUC 95% CI	0.949 (0.925–0.973)	0.849 (0.852–0.935)	0.940 (0.912–0.969)

A receiver operating characteristic (ROC) curve analysis was performed to obtain the optimal cut-off values for FT3, FT4, and FT3/FT4 ratio for the diagnosis of GD. The sensitivity and specificity of FT3 and FT4 level and FT3/FT4 ratio were estimated from the ROC curves. The positive predictive values (PPVs) and negative predictive values (NPVs) from the ROC curves for FT3, FT4, and the FT3/FT4 ratio were also calculated and compared. PPV: positive predictive values; NPV: negative predictive values; AUC: area under the curve; 95% CI: 95% confidence interval.

## Data Availability

The clinical data of the population used to support the findings of this study are available from the corresponding author upon request.
